# Sphingosine kinase 2 activates autophagy and protects neurons against ischemic injury through interaction with Bcl-2 via its putative BH3 domain

**DOI:** 10.1038/cddis.2017.289

**Published:** 2017-07-06

**Authors:** Dan-Dan Song, Tong-Tong Zhang, Jia-Li Chen, Yun-Fei Xia, Zheng-Hong Qin, Christian Waeber, Rui Sheng

**Affiliations:** 1 Department of Pharmacology and Laboratory of Aging and Nervous Diseases, Soochow University School of Pharmaceutical Science, Suzhou, China; 2 Department of Pharmacology and Therapeutics, University College Cork, Cork, Ireland; 3 School of Pharmacy, University College Cork, Cork, Ireland

## Abstract

Our previous findings suggest that sphingosine kinase 2 (SPK2) mediates ischemic tolerance and autophagy in cerebral preconditioning. The aim of this study was to determine by which mechanism SPK2 activates autophagy in neural cells. In both primary murine cortical neurons and HT22 hippocampal neuronal cells, overexpression of SPK2 increased LC3II and enhanced the autophagy flux. SPK2 overexpression protected cortical neurons against oxygen glucose deprivation (OGD) injury, as evidenced by improvement of neuronal morphology, increased cell viability and reduced lactate dehydrogenase release. The inhibition of autophagy effectively suppressed the neuroprotective effect of SPK2. SPK2 overexpression reduced the co-immunoprecipitation of Beclin-1 and Bcl-2, while Beclin-1 knockdown inhibited SPK2-induced autophagy. Both co-immunoprecipitation and GST pull-down analysis suggest that SPK2 directly interacts with Bcl-2. SPK2 might interact to Bcl-2 in the cytoplasm. Notably, an SPK2 mutant with L219A substitution in its putative BH3 domain was not able to activate autophagy. A Tat peptide fused to an 18-amino acid peptide encompassing the native, but not the L219A mutated BH3 domain of SPK2 activated autophagy in neural cells. The Tat-SPK2 peptide also protected neurons against OGD injury through autophagy activation. These results suggest that SPK2 interacts with Bcl-2 via its BH3 domain, thereby dissociating it from Beclin-1 and activating autophagy. The observation that Tat-SPK2 peptide designed from the BH3 domain of SPK2 activates autophagy and protects neural cells against OGD injury suggest that this structure may provide the basis for a novel class of therapeutic agents against ischemic stroke.

Sphingolipids, the major components in eukaryotic lipid bilayers and prokaryotic cell membranes, play important roles in cell survival and death.^1^ Sphingosine 1-phosphate (S1P) promotes cell survival and proliferation, while ceramide and sphingosine inhibit cell growth and induce cell apoptosis.^2^ Both isoforms of sphingosine kinases (SPK1 and SPK2) catalyze the phosphorylation of sphingosine to S1P, thereby controlling, together with S1P-degrading enzymes, the balance of sphingolipid species. SPK2 is likely to play a role in various diseases, such as cancer,^3, 4^ cardiovascular diseases^5, 6^ and inflammation.^7, 8, 9, 10^ Recent studies also demonstrate that SPK2 is involved in stroke, preconditioning and neuronal autophagy^11, 12, 13^ and may be a potential therapeutic target for the prevention and management of cerebral ischemia. But the mechanistic link between SPK2 and preconditioning or neuroprotection remains to be defined.

Autophagy is a dynamic process in eukaryotic cells that removes proteins or damaged organelles for recycling. In neurons, the autophagosomes at different maturation states in the cell soma are derived from distinct neuronal compartments, possibly to facilitate autophagosome degradation by fusion with lysosomes enriched in the soma. Surprisingly, neither rapamycin, a traditional autophagy inducer, nor nutrient deprivation induced neuronal autophagy as that in non-neuronal cells. This may indicate that the primary role of constitutive autophagy in neurons is to effectively turnover aging proteins and organelles to maintain homeostasis, rather than to mobilize amino acids under starvation.^14^ Autophagy may contribute to the neuroprotection induced by ischemic, hypoxic and isoflurane preconditioning (ISO).^15, 16, 17^ Various sphingolipids, including ceramide and S1P, have been shown to be involved in the regulation of autophagy.^18^ S1P produced by SPK1 overexpression in cell lines activates autophagy, possibly via suppression of mTOR activity and mild accumulation of Beclin-1.^19, 20^ Cytoplasmic S1P generated by SPK1 enhances autophagy flux in neurons,^21^ whereas the S1P lyase, a kind of S1P-metabolizing enzyme, downregulates autophagy.^22^ However, the contribution of SPK2 to autophagy within neurons remains to be elucidated. Our previous findings showed that the endogenous SPK2 isoform contributes to autophagy activation induced by ISO and hypoxic preconditioning.^17^ Interestingly, the SPK2 mediated autophagy and protection seemed to be S1P-independent, but possibly due to the disruption of Beclin-1/Bcl-2 interaction.^17^ SPK2 contains a 9-amino acid sequence similar to that seen in pro-apoptotic BH3-only proteins; indeed, SPK2 induces apoptosis in different cell types.^23^ BH3-only proteins such as Bad and BNIP3 have been shown to mediate autophagy by disrupting the interaction between Beclin-1 and Bcl-2 or Bcl-X_L_.^24, 25, 26^ We previously hypothesized that SPK2 might similarly induce autophagy by interaction with Bcl-2 via its BH3 domain.^17^

In the present study, we directly examined the effect of overexpressed SPK2 in primary cultured murine cortical neurons and HT22 neuronal cells to test the hypothesis that SPK2 could protect neural cells from ischemic injury by activating autophagy. Next we explored the mechanisms underlying autophagy activation induced by SPK2.

## Results

### SPK2 protects neurons from oxygen and glucose deprivation injury

**Figure 1:**
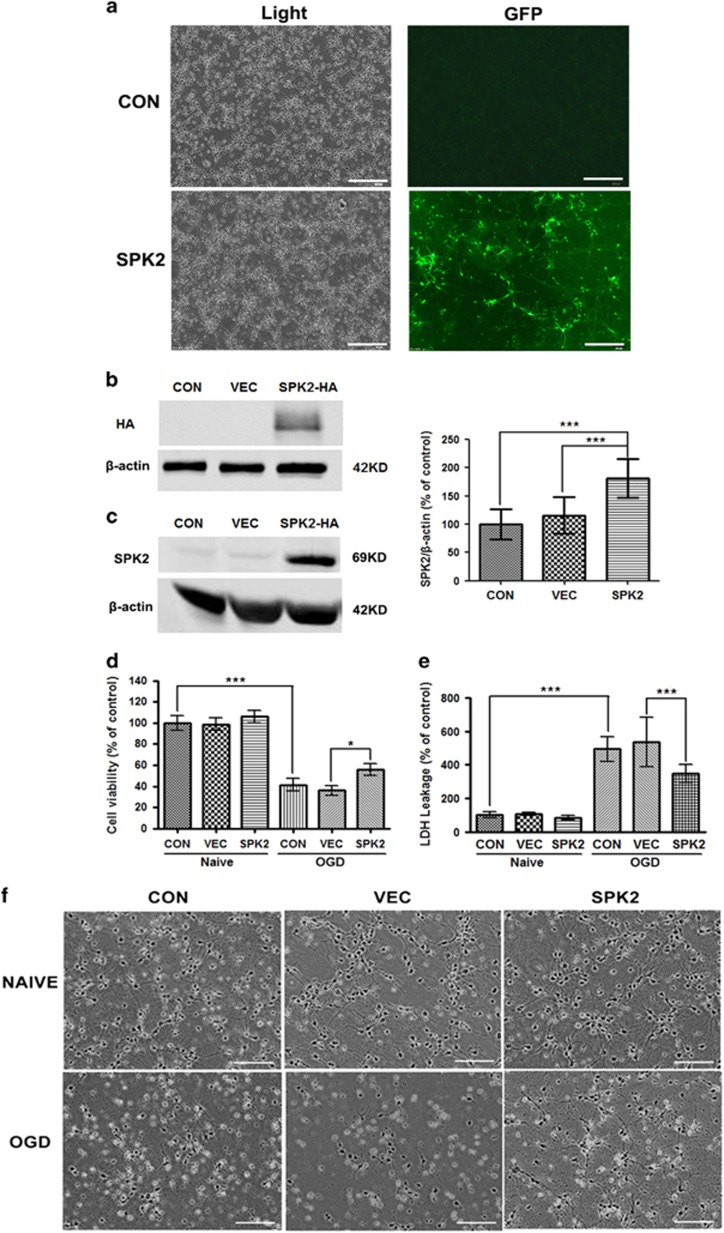
SPK2 overexpression protected primary cortical neurons from OGD injury. (**a**) Cortical neurons were infected with LV-SPK2 or LV-vector at DIV2. At DIV 7, the neurons were visualized with bright field, and the efficiency of infection was detected by GFP fluorescence. Scale bar=100 *μ*m. (**b** and **c**) Overexpression of SPK2 was induced by lentivirus gene infection. Neurons were harvested and subjected to western blot analysis against HA-tag (**b**) or SPK2 (**c**). (**d**) SPK2 protected cortical neurons from OGD injury. Cortical neurons were exposed to OGD for 4 h. The cell viability was examined with Cell Counting Kit-8 assay. (**e**) Cytotoxicity was examined with LDH assay after OGD treatment for 4 h. (**f**) The neuronal morphology was observed by optical microscope. Scale bar=100 *μ*m. Bar represents mean±S.D., *n*=3 independent experiments. *P<0.05, ***P<0.001

Neuronal and/or microvascular SPK2 have been shown to play a role in cerebral preconditioning.^11, 12, 13^ To examine whether SPK2 can directly protect against ischemic injury, we overexpressed SPK2 in primary murine cortical neurons; lentivirus-mediated SPK2 overexpression in cortical neurons was monitored with GFP immunofluorescence (Figure 1a). SPK2 and HA-Tag protein overexpression was confirmed by western blot analysis (Figures 1b and c). We also established a stable SPK2-overexpressing cell line (LV-SPK2-HT22 cells) by infecting HT22 cells with SPK2 lentivirus. SPK2, but not SPK1, was upregulated in LV-SPK2-HT22 cells (Supplementary Figure S1a), and the SPK2 activity significantly increased compared with LV-vector-HT22 cells (Supplementary Figure S1b). Cortical neuron cultures were then oxygen and glucose deprived for 4 h (OGD). Cell viability and cytotoxicity were determined using Cell Counting Kit-8 (CCK8) and lactate dehydrogenase (LDH) assay kits, respectively. OGD significantly decreased the viability and membrane integrity of cortical neurons, whereas SPK2-overexpressing neurons showed reduced cell death, as evidence by increased cell viability (Figure 1d), reduced LDH leakage (Figure 1e) and improved neuronal morphology (Figure 1f) compared with neurons infected with LV-vector.

### Overexpression of SPK2 induces autophagy activation

**Figure 2:**
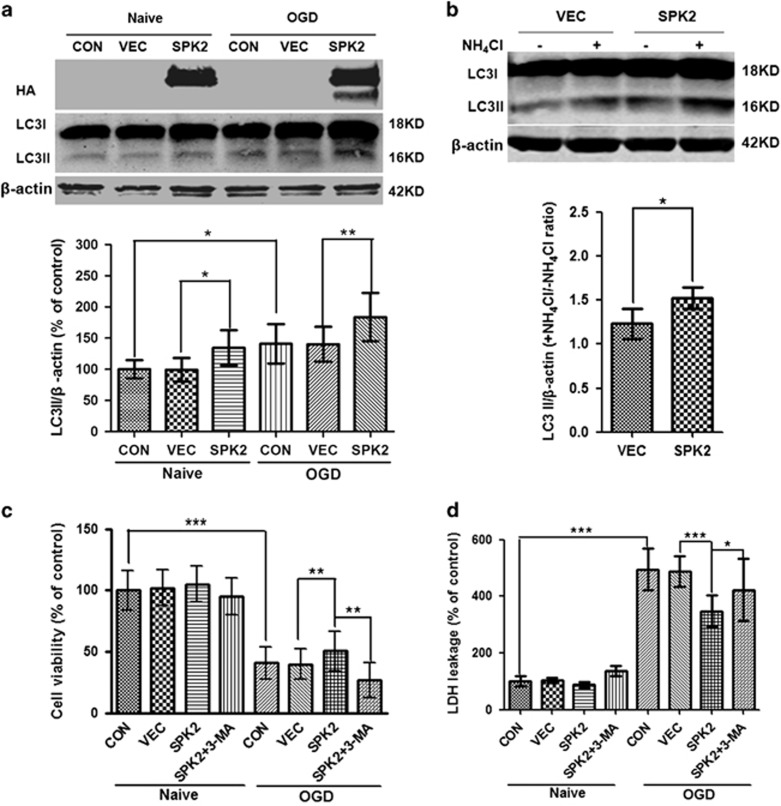
SPK2 overexpression induced autophagy activation in cortical neurons. (**a**) LC3II was upregulated in LV-SPK2-transfected neurons. Neurons were exposed to OGD for 4 h. After OGD treatment, cortical neurons transfected with LV-vector or LV-SPK2 were harvested and subjected to western blot analysis. (**b**) Autophagic flux was examined by comparing LC3II/actin with or without NH_4_Cl. Neurons were treated with NH_4_Cl (20 mM) for 24 h. (**c** and **d**) 3-MA abolished the neuroprotection induced by SPK2 overexpression. The neurons were treated with 3-MA (10 mM) for 24 h during OGD reperfusion. Cell counting kit-8 (**c**) and LDH release (**d**) was examined to determine cell viability and cytotoxicity. Bar represents mean±S.D., *n*=3 independent experiments. *P<0.05, **P<0.01, ***P<0.001

To expand on our previous observation that SPK2 may protect neurons through autophagy activation,^17^ we examined autophagic activity in SPK2-overexpressing neurons. The LC3II was increased after SPK2 overexpression compared with LV-vector-infected neurons. OGD upregulated LC3II, in agreement with published results,^16, 27, 28, 29, 30, 31^ while SPK2 overexpression further increased LC3II after OGD treatment (Figure 2a). We then examined the autophagic flux in SPK2-overexpressing neurons using ammonium chloride (NH_4_Cl), an inhibitor of lysosome-phagosome fusion.^32, 33^ The LC3II/actin ratio in the presence *versus* absence of NH_4_Cl increased in SPK2-overexpressing neurons compared with vector-infected neurons (Figure 2b), suggesting that overexpression of SPK2 enhanced autophagic flux. Similar to the results in cortical neurons, the LC3II in LV-SPK2-HT22 was much higher than that in LV-vector-HT22 cells (Supplementary Figure S2a), and SPK2 overexpression enhanced autophagy flux in LV-SPK2-HT22 cells as well (Supplementary Figure S2b).3-Methyladenine (3-MA) is a specific autophagy inhibitor that blocks the formation of autophagosomes.^34^ Pretreatment with 3-MA at a concentration known to block autophagy (10 mM)^16, 35, 36^ abolished the neuroprotection induced by SPK2 overexpression, as evidenced by reduced cell viability (Figure 2c) and increased LDH leakage (Figure 2d). All these results suggest that SPK2 mediates autophagy activation and contributes to the ischemic tolerance.

### Overexpression of SPK2 interferes with the interaction of Beclin-1 and Bcl-2

**Figure 3:**
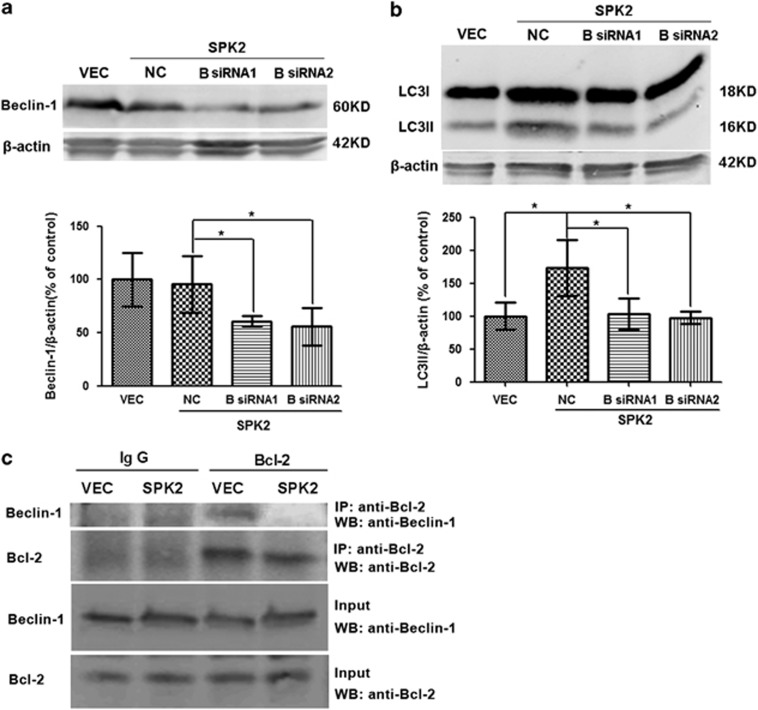
SPK2 disrupted Beclin-1/Bcl-2 complex to activate autophagy in cortical neurons. (**a** and **b**) Beclin-1 knockdown abolished SPK2-induced autophagy activation. Cortical neurons transfected with LV-vector or LV-SPK2 were further transfected with Beclin-1 siRNA on DIV6. Beclin-1 (**a**) and LC3 levels (**b**) were measured by western blotting. (**c**) SPK2 disrupted Beclin-1/Bcl-2 complex. Lysates were immunoprecipitated with anti-Bcl-2, separated by SDS-PAGE and subjected to blot analysis with the indicated antibody. Bar represents mean±S.D., *n*=3 independent experiments. *P<0.05. NC, negative control

Beclin-1, the mammalian homolog of yeast Apg6/Vps30, is a key regulator of autophagy.^37^ To test the hypothesis that the autophagy activation by SPK2 involves the Beclin-1 pathway, we examined the effect of siRNA-mediated Beclin-1 knockdown on autophagic activity. In SPK2-overexpressing neurons, Beclin-1 was effectively downregulated by two different Beclin-1 siRNAs (Figure 3a). Treatment with Beclin-1 siRNAs also markedly inhibited SPK2-induced LC3II upregulation (Figure 3b), suggesting that SPK2 activates autophagy in a Beclin-1-dependent manner. Beclin-1 interacts with Bcl-2 via its BH3 domain,^25, 38^ this interaction prevents Beclin-1 from activating autophagy, and autophagy is initiated when Beclin-1 is released from the Beclin-1/Bcl-2 complex.^39, 40^ To confirm our previous hypothesis that SPK2 can interfere with the interaction between Beclin-1 and Bcl-2,^17^ we further examined the co-immunoprecipitation of Beclin-1 and Bcl-2 in cortical neuron lysates. Beclin-1/Bcl-2 co-immunoprecipitation was reduced when SPK2 was overexpressed in cortical neurons (Figure 3c), confirming that SPK2 might disrupt the interaction between Beclin-1 and Bcl-2, resulting in autophagy activation.

### SPK2 interacts with Bcl-2

**Figure 4:**
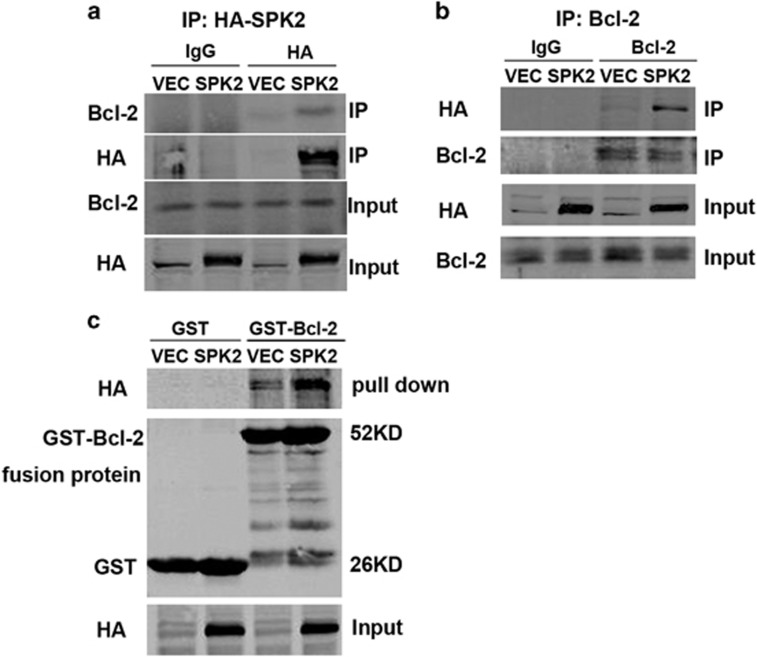
SPK2 interacts with Bcl-2 in HT22 cells. (**a**) Stable transfected LV-SPK2-HT22 cell lysates were immune-precipitated with anti-HA, separated by SDS-PAGE, and subjected to western blot analysis with anti-Bcl-2. (**b**) Cell lysates were immunoprecipitated with anti-Bcl-2, separated by SDS-PAGE, and subjected to western blot analysis with anti-HA. (**c**) GST-Bcl-2 or GST bound to glutathione-agarose, incubated with lysates of LV-HA-SPK2-transfected HT22 cells and subjected to western blot analysis with anti-HA. *n*=3 independent experiments

We then set out to test the hypothesis that SPK2 induces autophagy by interacting with Bcl-2 and examined the interaction of SPK2 and Bcl-2 by co-immunoprecipitation and GST pull-down analysis in lysates of SPK2-overexpressing HT22 cells. Bcl-2 was co-immunoprecipitated when HA-SPK2 was immunoprecipitated using HA antibodies, whereas Bcl-2 barely precipitated in cell lysates in the absence of HA-SPK2 (Figure 4a). Correspondingly, HA-SPK2 was co-immunoprecipitated when Bcl-2 was precipitated (Figure 4b). The interaction of SPK2 with Bcl-2 was further assessed by *in vitro* pull-down experiments using GST-Bcl-2 or GST alone bound to glutathione-agarose and lysates of SPK2- or vector-infected HT22 cells. The data demonstrated a specific interaction of SPK2 with GST-Bcl-2 but not with GST alone (Figure 4c). These results, taken together with the fact that SPK2 dissociates the complex between Beclin-1 and Bcl-2, further confirm that SPK2 interacts with Bcl-2 to release Beclin-1 from Beclin-1/Bcl-2 complex and to activate autophagy.

**Figure 5:**
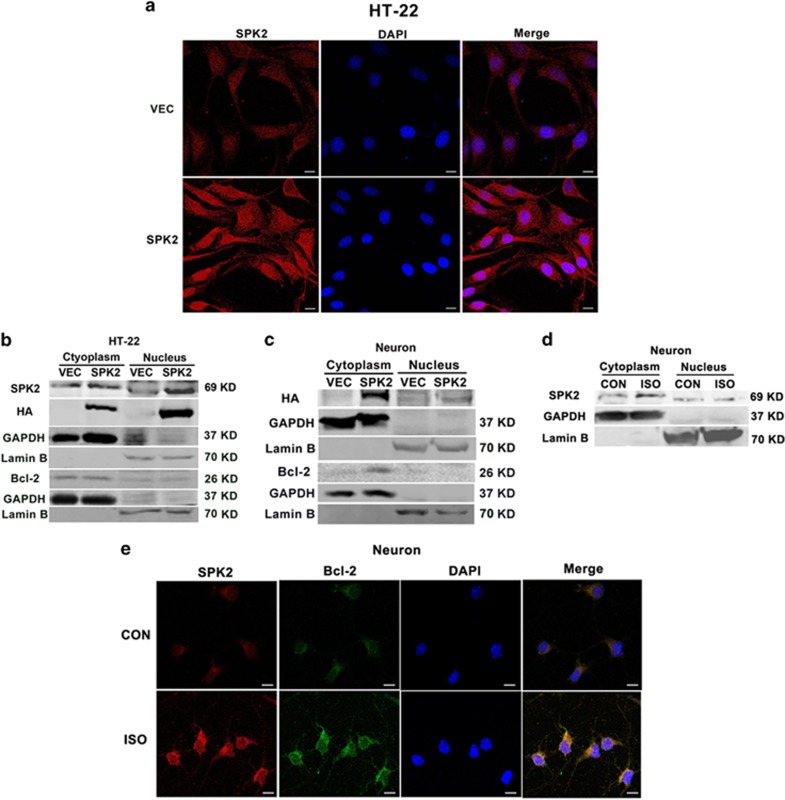
The distribution of SPK2 in the cytoplasm and nuclear fraction of neural cells. (**a**) LV-vector-HT22 and LV-SPK2-HT22 cells were fixed with 4% paraformaldehyde and processed for immunofluorescence. Representative images were stained with DAPI (blue) and antibody against SPK2 (red). Scale bar=10 *μ*m. (**b**) Nuclei and cytoplasm of LV-vector-HT22 and LV-SPK2-HT22 cells were extracted and subjected to western blot analysis. (**c**) Cortical neurons were infected with LV-SPK2 or LV-vector at DIV2. At DIV 7, the nuclei and cytoplasm of neurons were extracted and subjected to western blot analysis. (**d**) The distribution of SPK2 in the cytoplasm and nuclear fraction of neurons after isoflurane preconditioning (ISO). The neurons were exposed to 2% ISO for 30 min. Twenty-four hours later, the cytoplasmic and nuclear fractions of neurons were extracted and subjected to western blot analysis. (**e**) ISO increased the colocalization of SPK2 and Bcl-2 in the cytoplasm of neurons. Neurons were fixed with 4% paraformaldehyde and processed for immunofluorescence. Representative images were stained with DAPI (blue), and antibodies against SPK2 (red) and Bcl-2 (green). Scare bar =10 *μ*m. *n*=3 independent experiments

In neurons, SPK2 could be expressed in the cytoplasm, mitochondria and nucleus under physiological or pathological conditions.^41, 42, 43, 44^ Our previous finding showed that preconditioning mainly upregulates endogenous SPK2 in the cytoplasm. On the other hand, apoptosis-stimulating protein of p53-2 (ASPP2) could bind to Bcl-2 in the nucleus to prevent its translocation to the cytoplasm, thus contributing to Beclin-1-initiated autophagy.^45^ We then examined the cellular distribution of SPK2 and whether Bcl-2 translocates to the nucleus when SPK2 is overexpressed in neural cells. The immunofluorescence (Figure 5a) and western blot analysis (Figure 5b) showed that SPK2 was overexpressed in both the cytoplasm and nucleus of LV-SPK2-HT22 cells. In SPK2-overexpressing neurons, however, although SPK2 was also overexpressed in both cytoplasm and nucleus, its cytoplasmic content is much higher than that in nuclear fraction (Figure 5c). Bcl-2 was mainly expressed in the cytoplasm of both LV-SPK2-HT22 cells and SPK2-overexpressing neurons. There was no significant nuclear translocation of Bcl-2 under SPK2 overexpression. We also examined the cellular distribution of endogenous SPK2 in neurons after ISO, a noxious stimulus that could be applied conveniently to elicit neuroprotection *in vivo* and *in vitro*.^11^ The results showed that SPK2 was expressed in both cytoplasm and nucleus of control neurons. After ISO treatment, SPK2 was upregulated in the cytoplasm instead of nucleus (Figure 5d), consistent with our previous data of immunofluorescence assay showing that ISO increased the endogenous SPK2 mainly in a cytoplasmic manner.^17^ Importantly, ISO greatly increased the colocalization of endogenous SPK2 and Bcl-2 in cytoplasm (Figure 5e). All these results suggest that SPK2 might interact to Bcl-2 in the cytoplasm of neurons.

### Mutation in the putative BH3 domain of SPK2 (SPK2-L219A) prevents autophagy activation

**Figure 6:**
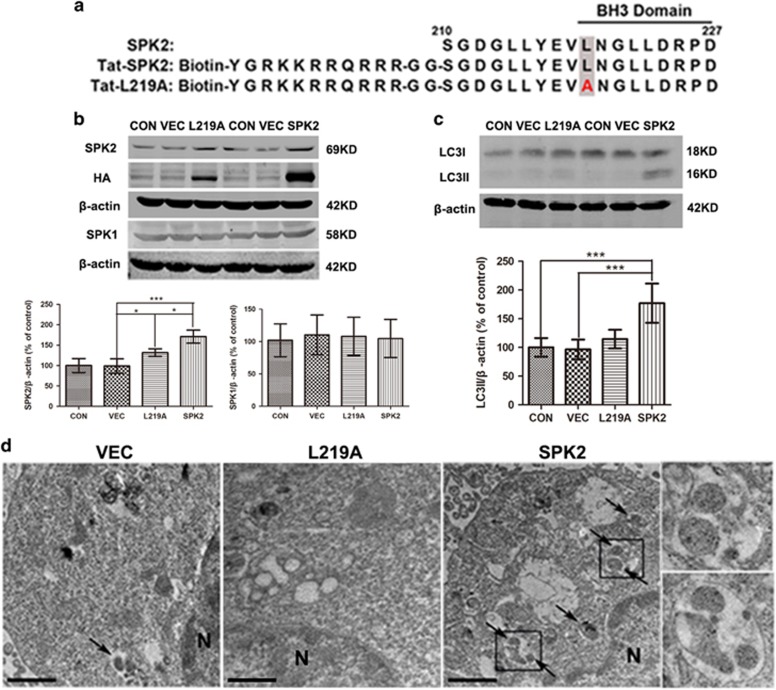
SPK2-L219A mutation prevents autophagy activation in HT22 cells. (**a**) Sequences of SPK2 amino acids 210–227, Tat-SPK2 and Tat-L219A peptide. (**b**) SPK2 and SPK1 expression in LV-L219A-HT22 and LV-SPK2-HT22 cells. The control, LV-vector, LV-SPK2 and LV-L219A-HT22 cells were harvested and subjected to western blot analysis. (**c**) LC3II was not upregulated in LV-L219A-HT22 as LV-SPK2-HT22 cells. (**d**) Electron microscopic images show increased number of double-membrane vacuolar structure in the LV-SPK2-HT22 but not in LV-L219A-HT22 cells. Scale bar=1 *μ*m. Arrows point to autophagosomes or autolysosomes. N: nucleus. Insets show the enlarged autophagosomes or autolysosomes taken from the boxed areas. Bar represents mean±S.D., *n*=3 independent experiments. *P<0.05, ***P<0.001

SPK2 contains a 9-amino acid motif found in the sequence of pro-apoptotic BH3-only proteins, and a Leu^219^ to Ala substitution in this sequence was previously shown to decrease the pro-apoptotic effect of overexpressed mutant SPK2 (Figure 6a).^23^ Because some BH3-only proteins (e.g., Bad and BNIP3) mediate autophagy by disrupting the interaction between Beclin-1 and Bcl-2 or Bcl-X_L_,^25, 26^ we then tested the hypothesis that the effect of SPK2 on autophagy is dependent on its BH3 domain. To do so, we mutated Leu^219^ to Ala and use the mutant SPK2 lentivirus to establish a stable LV-L219A-HT22 cell line. The apoptosis in the SPK2 overexpressed HT22 cells was examined by Hoechst staining. The basal apoptotic rate of LV-vector-HT22 cells is about 1.3%, and there is no significant difference in apoptotic rate among LV-SPK2-HT22, LV-L219A-HT22 and LV-vector-HT22 cells, suggesting that SPK2 overexpression did not induce significant apoptosis in neural cells (Supplementary Figure S3). Western blot quantification showed similar SPK1 levels in all cell lines (Figure 6b), but immunoreactivity for HA-Tag and for SPK2 were significantly higher in both LV-SPK2-HT22 and LV-L219A-HT22, compared with LV-vector-HT22 and control cells (Figure 6b). Furthermore, the LC3II was elevated in LV-SPK2-HT22 cells, but not in LV-L219A-HT22 cells (Figure 6c). Transmission electron microscope (TEM) was used to survey the extent of autophagosome formation in LV-SPK2-HT22 and LV-L219A-HT22 cells. HT22 cells infected with LV-vector appeared normal with relatively healthy-looking organelles and nuclei. The organelles and nuclei in LV-SPK2-HT22 and LV-L219A-HT22 cells also seemed normal without appreciable injury, but more double-membrane or multi-membrane vacuolar structures were found in the LV-SPK2-HT22 cells than that in LV-vector-HT22 and LV-L219A-HT22 cells (Figure 6d), providing morphological evidence that the putative BH3 domain of SPK2, and more specifically Leu^219^, plays an important role in SPK2-induced autophagy activation.

### Tat-SPK2 peptide induces autophagy activation

**Figure 7:**
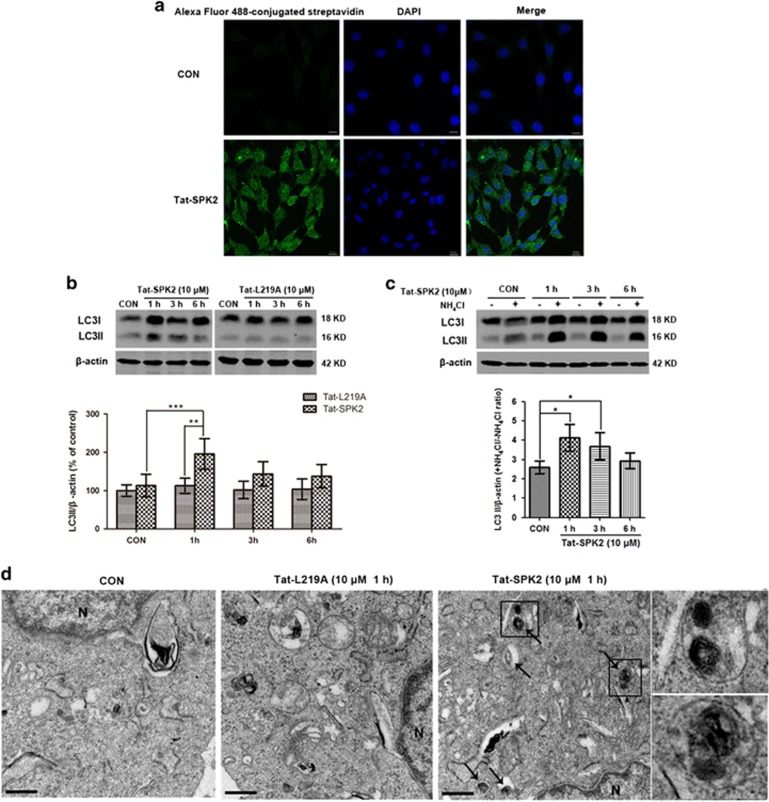
Tat-SPK2 peptide-induced autophagy in HT22 cells. (**a**) Intracellular staining pattern of biotin-conjugated Tat-SPK2 peptide. HT22 cells were treated with 10 *μ*M Tat-SPK2 or vehicle for 6 h, and stained with Alexa Flour 488-conjugated streptavidin. Scale bar=10 *μ*m. (**b**) Tat-SPK2 but not Tat-L219A upregulated LC3II. HT22 cells were treated with 10 *μ*M Tat-SPK2 or Tat-L219A peptide for indicated time. Then the cells were harvested and subjected to western blot analysis. (**c**) Autophagic flux was examined by comparing LC3II/actin with or without NH_4_Cl. HT22 cells were treated with 10 *μ*M Tat-SPK2 for indicated time, and then treated with 20 mM NH_4_Cl for 24 h. (**d**) Electron microscopic images show increased number of double-membrane vacuolar structure in HT22 cells treated with 10 *μ*M Tat-SPK2 peptide for 1 h but not in Tat-L219A peptide-treated HT22 cells. Scale bar=0.5 *μ*m. Arrows point to autophagosomes or autolysosomes. N: nucleus. Insets show the enlarged autophagosomes or autolysosomes taken from the boxed areas. Bar represents mean±S.D., *n*=3 independent experiments. *P<0.05, **P<0.01, ***P<0.001

We hypothesized that the BH3 domain of SPK2 might be sufficient to induce autophagy, and designed cell permeable Tat-SPK2 and Tat-L219A peptides composed of an 18-amino acid segment encompassing the BH3 domain of wild type or L219A-mutant SPK2, N-terminally conjugated to biotin^46, 47^ (Figure 6a). By imaging cells stained with Alexa Fluor 488–streptavidin conjugate, we confirmed that biotin-conjugated Tat-SPK2 was able to penetrate HT22 cell membranes (Figure 7a). Treatment with 10 *μ*M Tat-SPK2 peptide, but not Tat-L219A, increased the LC3II after 1h, indicating that Tat-SPK2 peptide did activate autophagy (Figure 7b). The LC3II in the presence *versus* absence of lysosomal protease inhibitor NH_4_Cl increased after Tat-SPK2 treatment at 1 and 3 h (Figure 7c), suggesting that autophagic flux was maintained up to 3 h after peptide treatment. The results of TEM showed that more double-membrane or multi-membrane vacuolar structures were found in HT22 cells treated with 10 *μ*M Tat-SPK2 peptide for 1 h than in the control group instead of Tat-L219A peptide (Figure 7d). All these results suggested that Tat-SPK2 peptide could activate autophagy.

### Tat-SPK2 peptide protects HT22 cells and primary neurons from OGD injury

**Figure 8:**
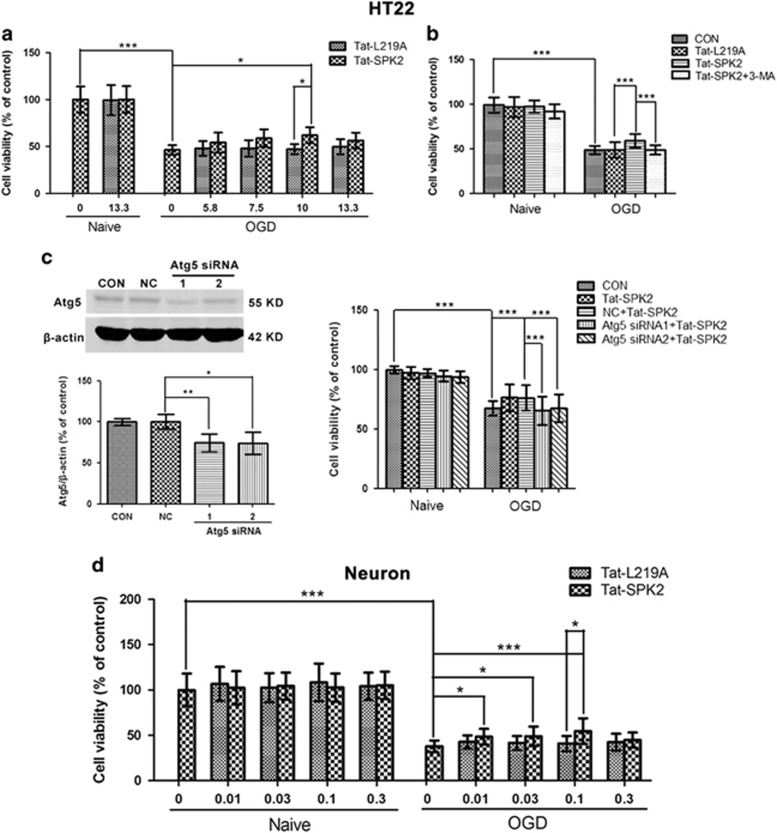
Tat-SPK2 peptide protected neural cells from OGD injury. (**a**) Tat-SPK2 peptide protected HT22 cells from OGD injury. HT22 cells were treated with Tat-SPK2 or Tat-L219A peptide at the indicated concentrations for 1 h, and then were exposed to oxygen glucose deprivation (OGD) for 6 h. The cell viability was examined by cell counting kit-8 assay. (**b**) 3-MA abolished Tat-SPK2-induced neuroprotection. Cells were treated with 10 *μ*M Tat-SPK2 or Tat-L219A peptide for 1 h, and then were exposed to OGD for 6h. Cells were treated with 2.5 mM 3-MA for 12 h during reperfusion. (**c**) Atg5 knockdown abolished Tat-SPK2-induced protection. HT22 cells were transfected with Atg5 siRNA using lipofectamine 2000. Atg5 was measured by western blotting. Seventy-two hours after transfection, cells were treated with 10 *μ*M Tat-SPK2 or Tat-L219A peptide for 1 h, and then were exposed to OGD for 6 h. (**d**) Tat-SPK2 peptide protected primary neurons from OGD injury. Primary neurons were treated with Tat-SPK2 or Tat-L219A peptide at the indicated concentrations for 3 h, and then were exposed to OGD for 4 h. Bar represents mean±S.D., *n*=3. *P<0.05, **P<0.01, ***P<0.001. NC, negative control

We then investigated whether Tat-SPK2 peptide treatment induced ischemic tolerance in HT22 cells and primary neurons. In HT22 cells, 10 *μ*M Tat-SPK2 peptide but not Tat-L219A significantly enhanced cell viability compared with OGD alone (Figure 8a). Notably, pretreatment with autophagy inhibitor 3-MA effectively abolished Tat-SPK2-induced protection (Figure 8b). Atg5 is a crucial gene involved in autophagosome biogenesis.^48, 49^ Two siRNAs directed against Atg5 were used to inhibit the induction of autophagy. Atg5 protein was efficiently reduced by Atg5 siRNAs compared with the negative control (NC). Knockdown of Atg5 could effectively abolish the neuroprotection induced by Tat-SPK2, further suggesting autophagy is involved in Tat-SPK2-mediated ischemic tolerance (Figure 8c). In primary neurons, Tat-SPK2 peptide at concentrations of 0.01 to 0.1 *μ*M also increased the cell viability compared with OGD alone or Tat-L219A group (Figure 8d). All these results suggested that Tat-SPK2 peptide could protect neural cells against OGD injury by autophagy activation.

## Discussion

**Figure 9:**
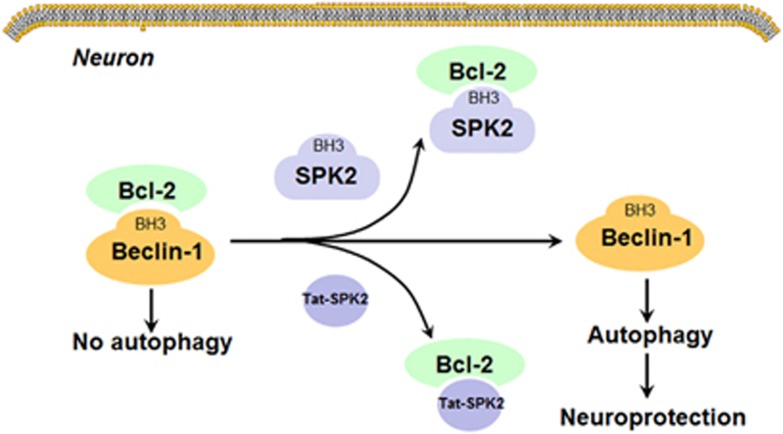
Proposed mechanism underlying autophagy activation induced by SPK2. Under normal conditions, Beclin-1 interacts with Bcl-2 via its BH3 domain to decrease the autophagy activity. Overexpression of SPK2 may displace Beclin-1 from Bcl-2 via its BH3 domain, leading to release of Beclin-1 and autophagy activation. Tat-SPK2 peptide containing BH3 domain of SPK2 might also bind to Bcl-2, and hence disrupt the interaction between Bcl-2 and Beclin-1 to induce autophagy. Autophagy then contributes to the neuroprotection induced by SPK2 or Tat-SPK2 peptide

The goal of the study was to expand on previous findings showing that SPK2 mediates ischemic tolerance and autophagy in cerebral preconditioning,^17^ and to explore the mechanism underlying autophagy activation induced by SPK2. There are many measurements to monitor autophagy, such as monitoring LC3II conversion, autophagy flux, autophagosomes or autolysomes, and detection of Atg proteins or other signals in the autophagy pathway, etc*.*^50^ In this study, we examined the LC3 levels, determined autophagy flux by using a lysosome inhibitor and used transmission election microscopy to observe autophagosomes and autolysosomes. In both primary murine cortical neurons and HT22 neuronal cells, SPK2 overexpression was associated with upregulated LC3II and enhanced autophagy flux. Knockdown of Beclin-1 inhibited SPK2-induced autophagy. SPK2 overexpression exerted neuroprotection against OGD injury in cortical neurons, and these effects were suppressed by autophagy inhibitor 3-MA. Our results indicated that SPK2 can interact with Bcl-2 via its BH3 domain, leading to the dissociation of Bcl-2 from Beclin-1 complex and to autophagy activation (Figure 9). Furthermore, the results of our experiments with Tat-SPK2 peptide suggested that agents mimicking the BH3 domain of SPK2 might be used therapeutically to induce autophagy and/or neuroprotection.

SPK2 can be upregulated by various preconditioning stimuli to mediate neuroprotection against cerebral ischemia^11, 12, 13^ and SPK2-mediated ischemic tolerance seems to involve autophagy.^17^ Data in this report confirmed previous findings and established causality by showing that SPK2 overexpression led to upregulated LC3II and enhanced autophagy flux, protected cortical neurons from OGD injury, whereas 3-MA, an autophagy inhibitor, effectively suppressed SPK2 elicited neuroprotection against OGD. All these results indicated that SPK2-induced autophagy activation contributes to its neuroprotection against OGD injury.

We previously hypothesized that SPK2-mediated autophagy during preconditioning was due to disruption of the interaction between Beclin-1 and Bcl-2, rather than to SPK2 catalytic activity.^17^ We now tested whether SPK2 overexpression directly activates autophagy via the Beclin-1 pathway. Our results showed that knockdown of Beclin-1 effectively suppressed the autophagy activation by SPK2, and SPK2 reduced the amount of co-immunoprecipitation of Beclin-1 and Bcl-2. In addition, both co-immunoprecipitation and GST pull-down analysis demonstrated that SPK2 directly interacted with Bcl-2. These results strongly suggested that SPK2 disrupts Beclin-1 /Bcl-2 complexes via its interaction with Bcl-2 to release free Beclin-1 and to activate autophagy.

Based on sequence analysis, SPK2 contains a 9-amino acid motif similar to the sequence in BH3-only proteins^51^ which contribute to cell apoptosis in some cancer cell lines.^23^ Beclin-1, via its BH3 domain, also interacts with Bcl-2 or Bcl-XL to regulate autophagy.^25^ Many BH3-only proteins are mediators of autophagy, since they can disrupt the interaction between Beclin-1 and Bcl-2/Bcl-XL to activate autophagy through the BH3 domains.^47, 52^ BNIP3, a BH3 domain-containing protein, can be upregulated by hypoxia-inducible factor-1*α* (HIF-1*α*) and displace Beclin-1 from Beclin-1/Bcl-2 or Beclin-1/Bcl-XL complexes, releasing Beclin-1 and regulating mitophagy.^26, 52, 53^ We thus proposed that SPK2 might activate autophagy via its BH3 domain by a mechanism similar to BNIP3. We site directed mutated Leu-219 in the BH3 domain of SPK2 to Ala (SPK2-L219A) and established stable L219A transfected HT22 cells.^23^ The L219A mutation not only showed reduced level of SPK2 expression, but also revealed compromised autophagy activity, as the LC3II and the amount of autophagosomes was obviously less in L219A-HT22 cells than that in SPK2-HT22 cells. These results implicated that the autophagy activation by SPK2 is dependent on its BH3 domain.

To further verify the role of SPK2′s BH3 domain in autophagy activation, we designed a cell permeable Tat-SPK2 peptide based on the sequence of the putative BH3 domain of SPK2.^23, 46^ Our data confirmed that Tat-SPK2 peptide could enter HT22 cells. As expected, Tat-SPK2 peptide instead of Tat-L219A could upregulate LC3II and increase the autophagy flux. Importantly, Tat-SPK2 peptide protected HT22 cells as well as primary neurons against OGD injury, while inhibition of autophagy effectively abolished Tat-SPK2-mediated ischemic tolerance. All these results implied that the BH3 domain of SPK2 is sufficient to activate autophagy and exert neuroprotection against ischemia.

Although SPK2 inhibits proliferation and enhances apoptosis through its BH3 domain in different cell types,^23^ recent reports also show that SPK2 plays a role in regulating cancer cell proliferation and migration^54, 55^ and SPK2 inhibitors may serve as the therapy for cancers.^56^ The discrepancy may be partly due to the expression level^57^ and cellular distribution of SPK2 in cells. The intracellular localization of SPK2 varies depending on cell types, activation state and/or density.^58, 59^ Although SPK2 is often a nuclear kinase,^58, 59, 60, 61, 62^ various groups also reported a cytoplasmic and mitochondrial localization of SPK2.^58, 60, 63, 64, 65^ In neurons, SPK2 can be expressed in the cytoplasm, mitochondria and nucleus under physiological or pathological conditions.^41, 42, 43, 44^ Recent study suggest that overexpressed SPK2 in nucleus is neurotoxic and promotes DNA double-strand breaks in cultured primary neurons.^44^ However, our data showed that SPK2 was overexpressed in both the cytoplasm and nuclei of the SPK2 overexpressed neurons and HT22 cells. On the other hand, Bcl-2 was mainly expressed in the cytoplasm of both LV-SPK2-HT22 cells and SPK2-overexpressing neurons. Importantly, ISO and hypoxic preconditioning, a noxious stimulus that could be applied conveniently to elicit neuroprotection *in vivo* and *in vitro*, could upregulate endogenous SPK2 to activate autophagy.^17^ ISO seems to increase the colocalization of endogenous SPK2 and Bcl-2 mainly in a cytoplasmic manner. From all these results, we assume that SPK2 might interact to Bcl-2 in the cytoplasm to activate autophagy. It remains to be elucidated whether the different distribution of SPK2 contributes to different signaling regulating autophagy and cell survival.

Our results are supported by several studies showing that mild endogenous SPK2 activation is one of the protective mechanisms in both cerebral ischemia and myocardial ischemia.^66, 67^ SPK2-mediated mechanism is involved in the protective effects of allicin against cerebral ischemia both *in vivo* and *in vitro*.^68^ SPK2 also contributes to the ischemic tolerance induced by cerebral or myocardial preconditioning.^11, 12, 13, 67, 69^ Overexpressed SPK2 in cardiomyocyte effectively inhibits apoptosis induced by hypoxia/reoxygenation.^69^ In agreement with these reports, no significant apoptosis was observed in SPK2-transfected HT22 cells in this study. Instead, SPK2 overexpression and Tat-SPK2 peptide containing BH3 domain of SPK2 protected neurons from OGD injury. In fact, BH3 domain has dual functions in apoptosis and autophagy.^47, 52^ BH3 proteins like Beclin-1^25, 70, 71^ and BNIP3^24^ contributes to survival rather than apoptosis under certain conditions, such as nutrient deprivation and hypoxia. The BH3 domains of hypoxia-induced BNIP3/BNIP3L could induce autophagy and cell survival by disrupting the Bcl-2/Beclin1complex without inducing cell death.^24^ We thus propose that SPK2, like low-affinity BH3 binding partners BNIP3 and Beclin-1, will not trigger cell death during hypoxia, possibly because the interaction between SPK2 and Bcl-2 is too weak in comparison with those of BAD or BIM to trigger apoptosis.^72^ On the other hand, it was reported that high-level overexpression of SPK2 inhibits cell survival and proliferation, while low-level overexpression of SPK2 promotes cell survival and proliferation.^57^ Thus the low level of SPK2 overexpression achieved in the present study (about twofold *versus* control) is similar to the effect of preconditioning to induce cell survival and protection.^17^ Furthermore, the dose of Tat-SPK2 peptide is also an important factor, as the lower dose of Tat-SPK2 might show stronger neuroprotection compared with higher dose. Tat-SPK2 peptide of 5.8–10 *μ*M in HT22 cells and 0.01–0.1 *μ*M in primary neurons showed a significant neuropotection against OGD injury. However, the higher dose of Tat-SPK2 (13.3 *μ*M in HT22 cells and 0.3 *μ*M in neurons) does not show changes compared with the L219A peptide. Tat-SPK2 peptide even shows toxicity at the dose over 20 *μ*M in HT22 cells and over 5 *μ*M in primary neurons (data not shown). All these results further support the notion that expression level of SPK2 is important for its function.

In conclusion, our present results demonstrated that SPK2 could activate autophagy to protect neurons from OGD injury. SPK2, via its BH3 domain, could dissociate Beclin-1/Bcl-2 complex by interacting with Bcl-2 to activate autophagy. Tat-SPK2 peptide designed from the BH3 domain of SPK2 could activate autophagy and protect neurons against OGD injury. SPK2 and Tat-SPK2 peptide promote neuronal survival through induction of autophagy, implying that SPK2 inducers, Tat-SPK2 peptide or agents acting on Beclin-1/autophagy pathway may provide candidate therapeutic agents against ischemic stroke. Further studies are required to investigate whether Tat-SPK2 peptide is effective in animal models of ischemic stroke.

## Materials and methods

### Cell culture

Primary cultured murine cortical neurons were prepared from the cortex of E15–17 mouse embryos.^15, 17^ Briefly, timed pregnant mice (Center of Experimental Animals of Soochow University, certificate No 20020008, Grade II) were killed by decapitation under 2% isoflurane. Cortices from embryos were dissected into pre-cooled PBS, and then digested in 1.25% trypsin at 37 °C for 15 min. The trypsin digestion was stopped by DMEM (Gibco) with 10% fetal bovine serum (FBS; Gibco, Waltham, MA, USA) and the cell suspension was treated with DNase I (Sangon Biotech, Shanghai, China) for 3 min. The cells were centrifuged at 500 × *g* for 5 min, and the supernatant was removed. The pellets were resuspended with neurobasal medium (NBM; Gibco) with 10% B27 (Gibco) and 25 *μ*M glutamate (Sigma, St. Louis, MO, USA). After filtration through 40 μm strainer, cells were plated on 0.1 mg/ml poly d-lysine (Sigma)-coated six-well (6 × 10^5^ cell/well) or 24-well (1.0 × 10^5^ cell/well) dishes. The medium was changed to NBM with 10% B27 and 0.5 mM glutamine (Sangon Biotech), 1% penicillin G and streptomycin after 24 h. Half the medium was changed every 2 days. Cortical neurons were used for experiments after 7–10 days *in vitro*.

HT22 mouse hippocampal neuronal cells were obtained from Shanghai Institute of Cell Biology (Shanghai, China). Cells were cultured in Dulbecco’s modified Eagle’s medium (DMEM; Gibco) supplemented with 10% FBS (Gibco), 100 U/ml streptomycin, 100 U/ml penicillin at 37 °C in 5% CO_2_/95% air.

### Lentivirus-mediated SPK2 overexpression

LV-SPK2 mouse-EGFP-HA-Puro (5 × 10^8^ TU/ml, GENEID 308589, NM020011) lentivirus was constructed by Shanghai Genechem Co. Ltd (Shanghai, China). On the second day *in vitro* (DIV2), cortical neurons were switched to NBM containing 10% B27, 0.5 mM glutamine and LV-SPK2 (MOI=10) or LV-vector (VEC) for 24 h. Afterwards, the medium was changed to the regular medium.^73^ The GFP immunofluoresence was observed at 7 days after transfection with a fluorescence microscope (Olympus; IX71, Tokyo, Japan).

To establish stably infected HT22 cells, the HT22 cells were infected with LV-SPK2 mouse-EGFP-HA-Puro, LV-SPK2-L219A mouse-EGFP-HA-Puro lentivirus (5 × 10^8^ TU/ml, HA-NM020011-mu1; Genechem, Shanghai, China) or LV-vector (MOI=10) for 24 h. Afterwards, the medium was changed to regular medium for 24 h. Cells were then selected with 2 *μ*g/ml puromycin (Sigma) for 2 weeks.^74^ The stable SPK2 or L219A infected HT22 cells were identified using GFP expression and western blot analysis.

### Oxygen glucose deprivation

Primary cultured cortical neurons or HT22 cells were washed three times with Hepes balanced salt solution (HBSS: NaCl 140 mM, KCl 3.5 mM, CaCl_2_ 1.7 mM, MgSO_4_ 12 mM, KH_2_PO_4_ 0.4 mM, NaHCO_3_ 5 mM, Hepes 10 mM; pH 7.2–7.4). Cultures were placed in a chamber (Billups-Rothenberg MC-101) filled with 95% N_2_ and 5% CO_2_ at 37 °C for 4 h (cortical neurons) or 6 h (HT22 cells). Upon removal of the cultures from the chamber, the growth medium was changed back to normal medium.^16^

### ISO model

Primary culture cortical neurons were exposed to 2% isoflurane (RWD Life Science, Shenzhen, China, R510–22, in 70% nitrogen and 30% oxygen) at 37 °C for 30 min in an airtight chamber. The neurons were handled 24 hours later.^17^

### Cell viability assay

Cell Counting Kit-8 (CCK8; Dojindo Laboratories, Tokyo, Japan) or LDH (Beyotime, Shanghai, China) assay was used to determine cell viability. For CCK8 assay, 24 h after OGD, 10 *μ*l CCK8 was added into each well of 96-well plates, which were incubated at 37 °C for 2 h. Optical density was measured at 450 nm using a plate reader (ELX 800; Bio-Tek, Winooski, VT, USA). For LDH assay, the neurons and culture medium were lysed in PBS containing 1% Triton X-100 at 37 °C for 30 min, respectively. The LDH activities in both the cell lysates and the culture mediums were assayed with the assay kit following the manufacturer’s instructions. LDH leakage was calculated as follows: LDH leakage (%)=LDH culture medium/(LDH culture medium+LDH cell lysates) × 100%.^73^

### Sphingosine kinase 2 activity assay

SPK2 activity was determined using the fluorescent substrate NBD-sphingosine (avanti Polar Lipids; 810205P).^12, 75^ HT22 cells were collected in lysis buffer (Hepes 50 mM, pH 7.4, KCl 10 mM, glycerol 20%, dithiothreitol 2 mM, NaF 15 mM, semicarbazide 2 mM, EDTA-free complete protease inhibitor (Roche, Basel, Switzerland)). Twenty-five micrograms of lysate protein reacted with reaction mixture (Hepes 50 mM, pH 7.4, MgCl_2_ 15 mM, glycerol 10%, ATP 10 mM, NaF 15 mM, semicarbazide 2 mM, NBD-sphingosine 10 *μ*M and KCl 1 M (for SPK2 specificity^12^)). After 1 h, the reaction was stopped by 50 *μ*l 1 M K_3_PO_4_ (pH 8.5). Then 250 *μ*l CHCl_3_/CH_3_OH (2:1) was added and the lysates were centrifuged at 15000 *× g* for 1 min. One hundred microliters upper aqueous phase was incubated with 50 *μ*l dimethylformamide and NBD fluorescence was read by Multiskan Spectrum Microplate Reader (TECAN Infinite M1000 Pro, Mannedorf, Siwtzerland), excitation at 485 nm, emission at 538 nm).

### Western blot analysis

Cells were rinsed twice with cooled PBS and lysed in a buffer containing Tris-HCl (pH 7.4) 10 mM, NaCl 150 mM, 1% Triton X-100, 1% sodium dexoxycholate, 0.1% SDS, EDTA 5 mM and EDTA-free complete protease inhibitor (Roche). Protein concentrations were determined by BCA assay. Equal amounts (10–30 *μ*g) of total protein extracts were separated by SDS-PAGE and transferred to nitrocellulose membranes. Western blot analysis was used to measure the levels of SPK2 (1 : 200; Abgent, San Diego, CA, USA, AP7238a), HA-Tag (1:1000; CST3724S), SPK1 (1 : 1000, Abgent AP7237c), LC3 (1 : 1000; Abcam, Cambridge, UK, ab62721), Beclin-1 (1 : 1000, Santa Cruz, Dallas, TX, USA, sc-11427). Expression levels were normalized to *β*-actin (1 : 10000; Sigma A5441).

### Hoechst staining

Cells were fixed with 4% paraformaldehyde for 15 min and the nuclei were stained with Hoechst 33342 for 15 min. Fluorescence was observed with a fluorescence microscope (Olympus, IX71). Cells undergoing apoptosis were characterized by brightly stained, condensed nuclei. The percentages of apoptotic cells were counted in four random fields.

### SPK2 and/or bcl-2 immunofluorescence

Cells were fixed with 4% paraformaldehyde for 15 min and incubated with PBS containing 0.1% Triton X-100 for 30 min. After blocking with 1% BSA for 1 h, cells were incubated with antibodies against SPK2 (1:100) and/or Bcl-2(1:20; Santa Cruz sc-7382) at 4 °C for 24 h, and with anti-rabbit IgG(H+L) secondary antibody, Dylight 633 (1:500; Invitrogen) and/or anti-mouse IgG (H+L) secondary antibody, DyLight 488 (1:500; Invitrogen) for 2 h. Then the cells were stained with DAPI (1:10000; sigma D9564) for 10 min. Images of fluorescence were acquired using a laser scanning confocal microscopy (ZEISS LSM710, Oberkochen, Germany).^17^

### Nuclear extraction

Cells were centrifuged at 1500 × *g* for 5 min at 4 °C and lysed in buffer A (sucrose 1 M, CaCl_2_ 0.1 M, MgAc 1M, EDTA, DTT 100 nM, NP40 0.5%, EDTA-free complete protease inhibitor (Roche)). The lysates were then centrifuged at 600 × *g* for 15 min to separate the cytoplasmic fraction of the cell extract. The pellets were resuspended in buffer B (sucrose 1 M, CaCl_2_ 0.1 M, MgAc 1 M, EDTA 250 mM, DTT 100 nM), and centrifuged at 600 × *g* for 10 min. The pellets were taken as the nuclear fraction.^76^ Lamin B (Santa Cruz sc6216) and GAPDH (Abcam, ab8245) were used as nuclear and cytoplasmic markers.

### Co-immunoprecipitation

Cells were harvested and lysed in a buffer containing Tris-HCl 50 mM (pH 7.4), NaCl 150 mM, 0.05% sodium deoxycholate, 1% NP40 and EDTA-free complete protease inhibitor (Roche). The lysates were precleaned with protein G-agarose (Roche) for 1 h, incubated with antibody (Bcl-2, Santa Cruz sc-7382; HA-Tag, CST3724S) or IgG (normal mouse IgG, Santa Cruz sc-2025, normal rabbit IgG, Santa Cruz sc-2027) overnight. After washing away unbound antibody, lysates were incubated with protein G-agarose for 4–8 h.^17^ The immunoprecipitates were analyzed by western blotting using antibody against HA, Bcl-2 (Santa Cruz sc-492) or Beclin-1.

### GST pull-down

Glutathione-agarose (Santa Cruz sc-2009) beads were washed with 1% Triton X-100 PBS, and were incubated with GST (Flarebio, Wuhan, China, CSB-RP101744Ba) or Bcl-2 GST fusion protein (PTG, Wuhan, China, ag3508) for 1 h at 4 °C under constant mixing. After washing away unbound proteins three times with pre-cooled lysis buffer, the GST-bead complex was incubated overnight with cell lysates.^77^ The bound proteins eluted from the beads were subjected to western blot analysis using anti-HA or anti GST antibody (1 : 1000, Santa Cruz sc-138).^78^

### Electron microscopy

HT22 Cells were harvested and fixed in 2.5% gluteraldehyde in 0.1 M phosphate buffer (pH 7.4) at 4 °C. The cell pellets were post-fixed with 1% Osmium tetroxide, dehydrated through acetone and then immersed in resin. After hardening, the blocks were sectioned at 50 nm thickness and stained with lead citrate. Then the autophagosomes or antolysosomes were viewed on a Transmission Electron Microscope (JEOL JEM 1230,Tokyo, Japan). Autophagosomes were defined as double- or multiple-membrane structures surrounding cytoplasmic constituents, while autolysosomes were defined as single membrane structures containing cytoplasmic components.^79^ At least 10 fields of view of each sample were captured. Three independent experiments with different samples were analyzed.

### Peptide synthesis and treatment

Tat-SPK2 peptide and Tat-L219A peptide (Figure 6a) were synthesized by Chinese Peptide Company (Hangzhou, China, purity 95%). Tat-SPK2 peptide sequence consisted of 11 amino acids from the Tat protein transduction domain (PTD),^46^ a GG linker, and the 18 amino acids derived from the SPK2 210–227.^23^ The control peptide, Tat-L219A, consisted of PTD, a GG linker and 18 amino acids of SPK2 210–227 containing one substitution, L219A. The Tat-SPK2 and Tat-L219A peptides were dissolved in double distilled water and then diluted to the working solution with complete medium. To determine the autophagy activity, HT22 cells were pretreated with the peptides at 10 *μ*M for 1–6 h. For cell viability analysis, HT22 cells were pretreated with the peptides at the indicated concentrations for 1 h before OGD and during the whole OGD period. Primary neurons were pretreated with the peptides for 3 h, and then exposed to OGD for 4 h. The peptide was removed during OGD episode.

### Peptide entry assay

HT22 cells were treated with 10 *μ*M biotinylated Tat-SPK2 for 6 h. Cells were fixed with 4% paraformaldehyde for 10 min at room temperature. Then HT22 cells were permeabilized with 0.1% Triton X-100 and stained with Alexa Fluor 488-conjugated streptavidin for 1 h.^47^ Fluorescence was observed by a laser scanning confocal microscope (ZEISS LSM710).

### Drug treatment

Cortical neurons or HT22 cells were treated with autophagy inhibitor (Sigma; M9281) for 12–24 h following OGD.^16, 34, 36^ Neurons or HT22 cells were treated with an lysosomal inhibitor NH_4_Cl (Greagent G17391B) 20 mM for 24 h.^32, 33, 74^

### siRNA

Small-interfering RNAs against Beclin-1 (siRNA1, sense: 5′-GGAGUGGAAUGAAAUCAAUTT-3′, antisense: 5′-AUUGA -UUUCAUUCCACUCCTT-3′ siRNA 2, sense: 5′-GAUCCUGG -ACCGGGUCACCTT-3′, antisense: 5′-GGUGACCCGGUCCAGGAUCTT-3′)^17^ or Atg5 (siRNA1, sense: 5′-GCGGUUGAGGCUCACUUUATT-3′, antisense: 5′-UAAAGUGAGCCUCAACCGCTT-3; siRNA 2, sense: 5′-CCAUCAACCGGAAACUCAUTT-3′, antisense: 5'-AUGAGUUUCCGGUUGAUGGTT-3′) were synthesized by Genepharma (Shanghai, China). Transfection of Beclin-1 siRNA (40 nM) in cortical neurons was performed using lipofectamine 2000 (Invitrogen) on day 6 *in vitro* (DIV6)^80^ according to the manufacturer’s instructions. HT22 cells were transfected with Atg5 siRNA (80nm) using lipofectamine 2000. The cells were also transfected with a control scrambled RNA targeting a sequence not sharing homology with the *Mus Musculus* Genome (negative control, NC). The suppression of Beclin-1 or Atg5 expression was confirmed with western blotting.

### Statistical analysis

The data were expressed as mean±S.D. The significance between groups was determined with one-way ANOVA, followed by the Newman–Keuls test for *post hoc* analysis.
