# Supplementary material for: Sphingosine kinase 2 activates autophagy and protects neurons against ischemic injury through interaction with Bcl-2 via its putative BH3 domain
Source: Cell Death Dis. 2017 Jul 6;8(7):e2912–. doi: 10.1038/cddis.2017.289 (PMC5550846; doi:10.1038/cddis.2017.289)
Supplement: Supplementary Figure Legends [file cddis2017289x1.doc]

**Supplement**

**Figure S1.**  Increased SPK2 expression in stable transfected LV-SPK2-HT22 cells. (a) HT22 cells were infected with LV-SPK2 or LV-vector, and selected by 2µg/ml puromycin for two weeks to establish LV-SPK2, LV-vector (VEC)-HT22 cells. SPK2, HA and SPK1 levels were measured by Western blotting. (b) Sphingosine kinase 2 activity was measured using NBD-sphingosine as indicated in the method. Bar represents mean±SD, *n*=3 independent experiments. * *P* <0.05, *** *P* <0.001.

**Figure S2.**  SPK2 overexpression induced autophagy activation in HT22 cells. (a) The control, stable transfected LV-SPK2, LV-VEC-HT22 cells were harvested and subjected to Western blotting. (b) LV-SPK2-HT22 cells were treated with NH_4_Cl (20mM) for 24h. LC3 levels were measured by Western blotting. Bar represents mean ± SD, *n*=3 independent experiments. * *P* <0.05, ** *P<*0.01.

**Figure S3**. No significant apoptosis in SPK2 overexpressed HT22 cells. (a). LV-vector-HT22, LV-L219A-HT22, LV-SPK2-HT22 cells were fixed with 4% paraformaldehyde, stained with Hoechst 33342 and observed with a fluorescence microscope. Scale bar=100 μm. (b). Quantitative analysis of the apoptotic cells. Bar represents mean ± SD, *n* = 3.
