# Supplementary figures and images for: Sphingosine kinase 2 activates autophagy and protects neurons against ischemic injury through interaction with Bcl-2 via its putative BH3 domain
Source: Cell Death Dis. 2017 Jul 6;8(7):e2912–. doi: 10.1038/cddis.2017.289 (PMC5550846; doi:10.1038/cddis.2017.289)

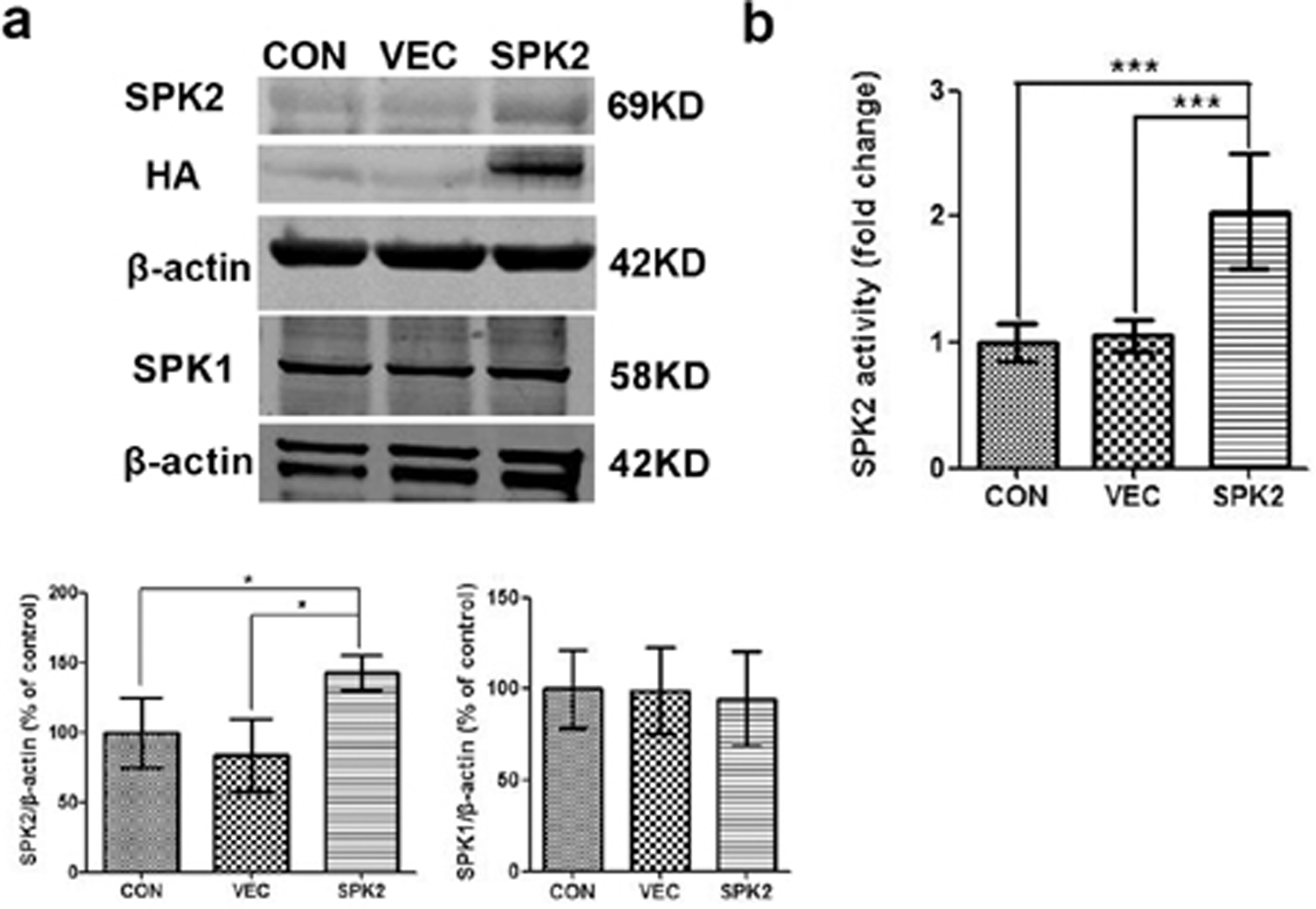

Supplement: Supplementary Figure S1 [file cddis2017289x2.tif]

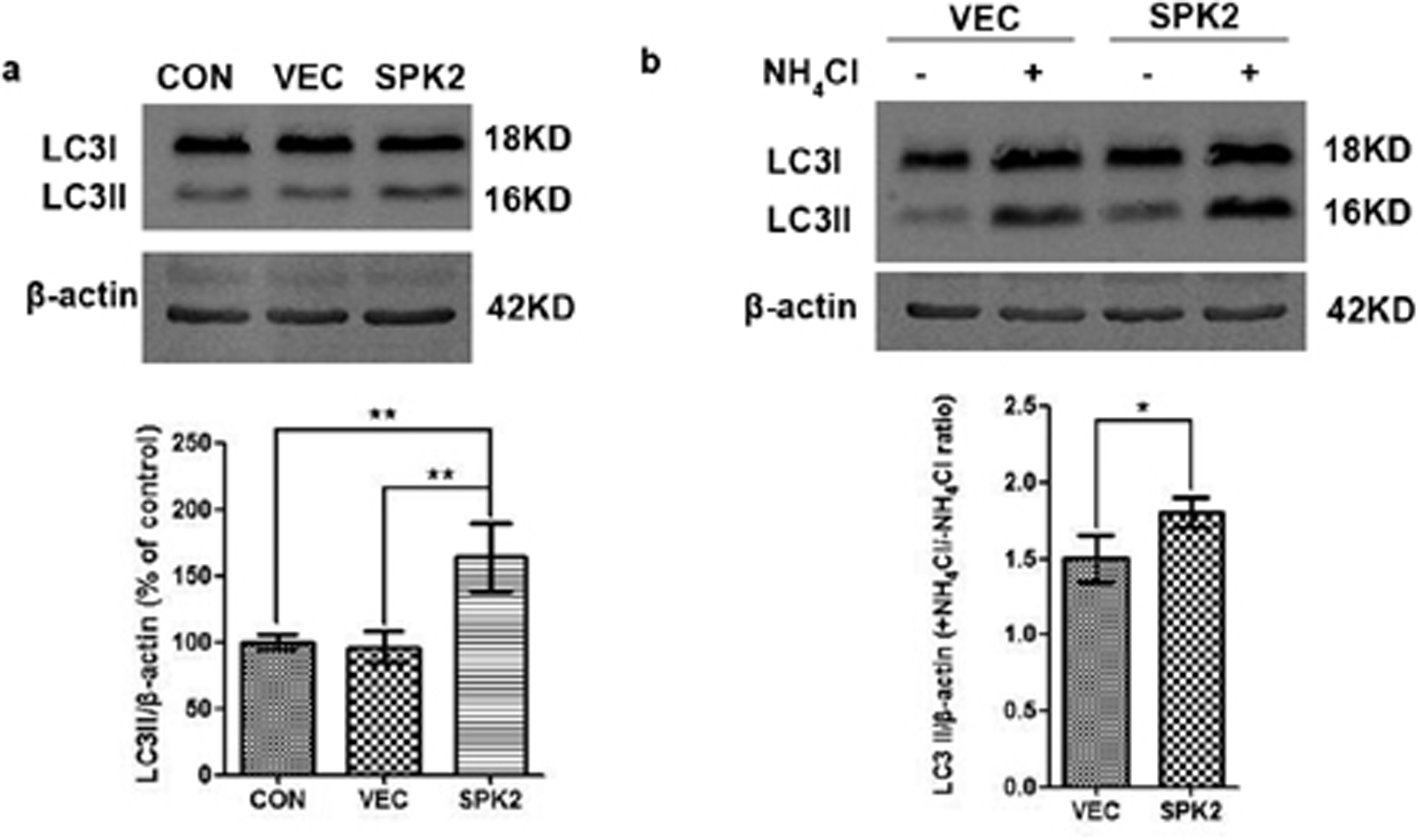

Supplement: Supplementary Figure S2 [file cddis2017289x3.tif]

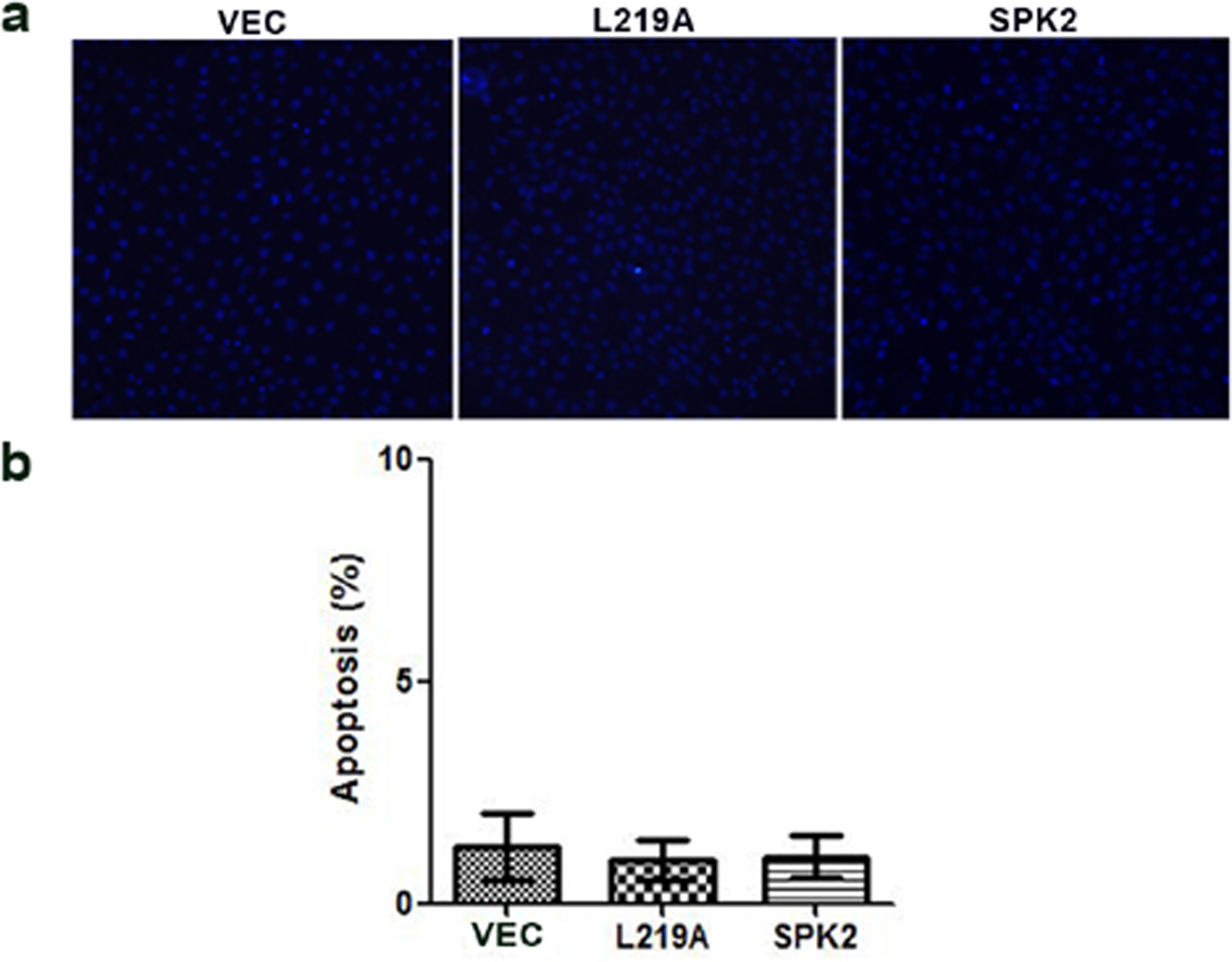

Supplement: Supplementary Figure S3 [file cddis2017289x4.tif]
